# Overendocytosis of superparamagnetic iron oxide particles increases apoptosis and triggers autophagic cell death in human osteosarcoma cell under a spinning magnetic field

**DOI:** 10.18632/oncotarget.14114

**Published:** 2016-12-23

**Authors:** Shaohua Du, Jingxiong Li, Chonghua Du, Zhongming Huang, Guangnan Chen, Weiqi Yan

**Affiliations:** ^1^ Department of Orthopedic Surgery, The Second Affiliated Hospital, School of Medicine, Zhejiang University, Hangzhou, 310008, China; ^2^ Department of Obstetrics and Gynecology, Third Affiliated Hospital of Guangzhou Medical University, Guangzhou, 510150, China; ^3^ School of Economics, Dongbei University of Finance and Economics, Dalian, 116025, China; ^4^ Department of Orthopaedic Surgery, Xiaoshan Chinese Medical Hospital, Hangzhou, 311201, China

**Keywords:** superparamagnetic iron oxide particles, apoptosis, autophagy, osteosarcoma, spinning magnetic field

## Abstract

The toxicity of superparamagnetic iron oxide nanoparticles (SPIONs) is still a vital topic of debate and the mechanisms remain unclear. In the present study, overdose SPIONs could induce osteosarcoma cell death and the effects were exaggerated when combined with spinning magnetic field (SMF). In the combination group, mitochondrial transmembrane potential decrease more obviously and reactive oxygen species (ROS) was found to generate much higher in line with that of the apoptosis ratio. Meantime, amount of autophagy was induced. Inhibiting the autophagy generation by 3-methyladenine (3-MA) increase cell viability but decrease the caspase 3/7 and caspase 8 activities in combination groups, and inhibiting apoptosis took the same effect. In the end, the SPIONs effects on xenograft mice was examed by intratumoral injection. The result showed that the combination group could greatly decrease the tumor volume and prolong the lifespan of mice. In sum, the result indicated that overdose SPIONs induced ROS generation, and excessive ROS induced by combination of SPIONs and SMF contribute to autophagy formation, which play a apoptosis-promoting role that formed as a platform to recruits initiate the caspase activities.

## INTRODUCTION

Today, nanotechnology and engineered nanoparticles (NPs) are widely used in healthcare and life sciences fields, medical and biotechnological applications areas [1, 2]. Usually the diameter of NPs range from 1 to 100 nm, and partially due to their smaller size, lager surface-to-volume ratio and increased display reactivity [3], NPs always display unique physicochemical characteristics. The diverse potential of NPs make it highly possible that the exposure ratio of public to NPs will increase dramatically in the next few decades. Therefore, to understand the potential adverse effects of NPs has become a priority for regulating the nanotechnologies protection [4].

To be as a type of novel material, Magnetic iron oxide nanomaterials, especially the Superparamagnetic iron oxide nanoparticles (SPIONs), have raised extensive attention because of their great biocompatibility and biodegradability [5, 6]. SPIONs exhibit magnetic behavior only there is a magnetic field, so they are of great interest for applications *in vivo* and *in vitro* [7, 8]. Ferroferric oxide (Fe3O4) nanoparticles, one example of the type of material, display great potential for medical applications. SPIONs are used to be a contrast agent in magnetic resonance imaging (MRI) [9, 10] and ultrasonography [11, 12], to damage tumors in alternating magnetic field through hyperthermia [13, 14] and to be as a carriers in drug delivery systems [15]. In the case of thermal therapy, the particles convert the energy of magnetic field into heat through the Brownian and Neel relaxation [16]. Although it is always emphasized that the SPIONs is low toxicity, some recent studies have showed that these NPs might induce cellular apoptosis or other responses [17]. It was reported that, the NPs can display cytotoxicity and apoptosis in non-small lung cancer cells but only induce limited toxicity to cervical cancer cells [17].Several mechanisms for SPIONs-induced cell and tissue injury are supported by limited experimental evidence. One hypothesis that are the most developed for nanoparticle toxicity is reactive oxygen species (ROS), which is believed to induce damage in protein, DNA and tissue [18].

In addition to apoptosis, another type of cell response-macroautophagy, which referred as autophagy hereafter, is generally evoked as a cytoprotective mechanism that under exposure to drugs, nutrient deprivation or hypoxia [19, 20]. Autophagy involves several steps of lysosomal degradation process, in which cells can eliminate damaged organelles and degrade aged proteins. However, in contrast to the survival-induced autophagy, many investigations have indicated that autophagy can also contribute to cytotoxicity and cell death. Therefore, whether autophagy plays a prosuvival or prodeath role under some circumstances, such as overdoes SPIONs administration combined with magnetic field, are needed to be investigated.

Until now, studies on the biological effects of SPIONs always focus on drug delivery and thermal therapy in alternative magnetic field; however, sedum has pay attention to the toxicity of the SPIONs their own. Moreover, the mainly disadvantage of alternative magnetic field induced thermal energy is inhomogeneous heating and side reaction of harm to the normal nearby tissue. The physical rotation and vibration of the SPIONs which may also induce toxicity was ignored. Therefore, we employed an new type of magnetic field-spinning magnetic field (SMF), which a cylinder shaped magnet spins on its axis, and generate a magnetic field that is also spins on the same axis, can combined with the SPIONs without the thermal effect production.

In this study, we synthesized SPIONs and assessed their cytotoxicity of inducing ROS and autophagy as well as apoptosis and mitochondrial disruption in osteosarcoma cell lines *in vitro* and *in vivo*, combined with present and absent of SMF, to investigate the mechanism of cell death and to develop another method for the tumor therapy.

## RESULTS

### Synthesis and characterization of SPIONs

**Figure 1:**
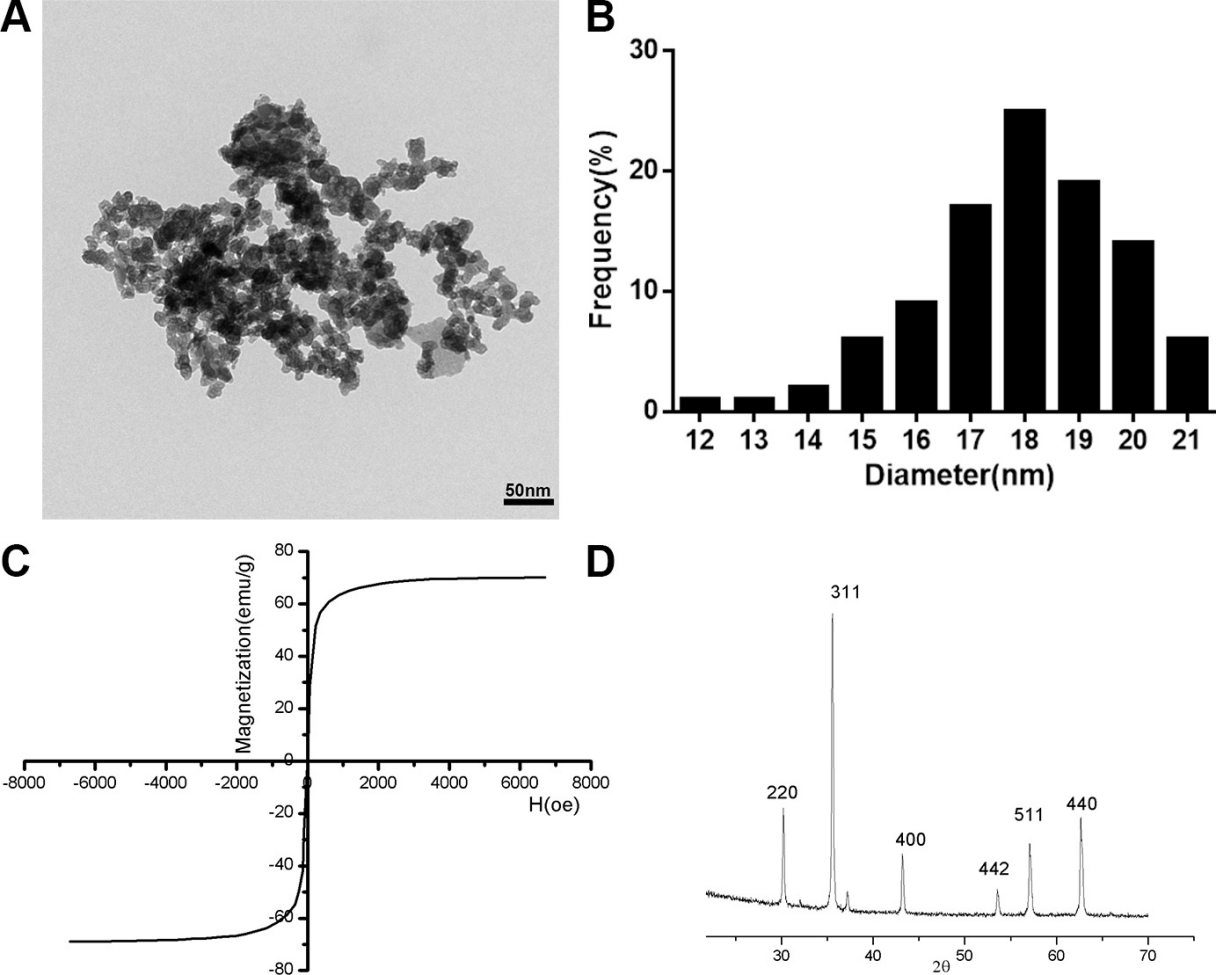
Characterization of the SPIONs (**A**) TEM images of SPIONs (scale bar, 50 nm). (**B**) Size distributions measurements of the SPIONs. (**C**) Magnetization curves. Magnetization curves were recorded on a vibrating sample magnetometer. (**D**) X-ray diffraction patterns of SPIONs. results reveal the crystal structure of the SPIONs and no characteristic peaks of impurities were detected.

Particles of SPIONs were synthesized and characterized as described previously. To assess the particle shape and size, synthesized SPIONs were analyzed by TEM. Figure 1A showed that the SPIONs were spherically shaped or slightly elliptical shape with a narrow size distribution. The size ranged from 12 nm to 21 nm showing a mode value of 18 nm (Figure 1B). magnetic measurement on SPIONs indicated that the particles are superparamagnetic at room temperature, as showed in Figure 1C, there was no coercive force in hysteresis loop of SPIONs, the magnetization of the particles approached the saturation values (67.9 emu/g) under a large external field, a little less than the bulk magnetite of 84 emu/g [21]. Figure 1D is a representative XRD patterns of SPIONs. The relative intensity of diffraction pattern and interplanar spacing well matched with standard Fe_3_O_4_ nanoparticles [22]. According to Scherrer's formula [23], the average particle diameter is consistent with that of the TEM assay, showed that the SPIONs were crystalline. From the characterization, we can see that the SPIONs prepared in our experiment were of good quality.

### Cytotoxicity of the SPIONs

**Figure 2:**
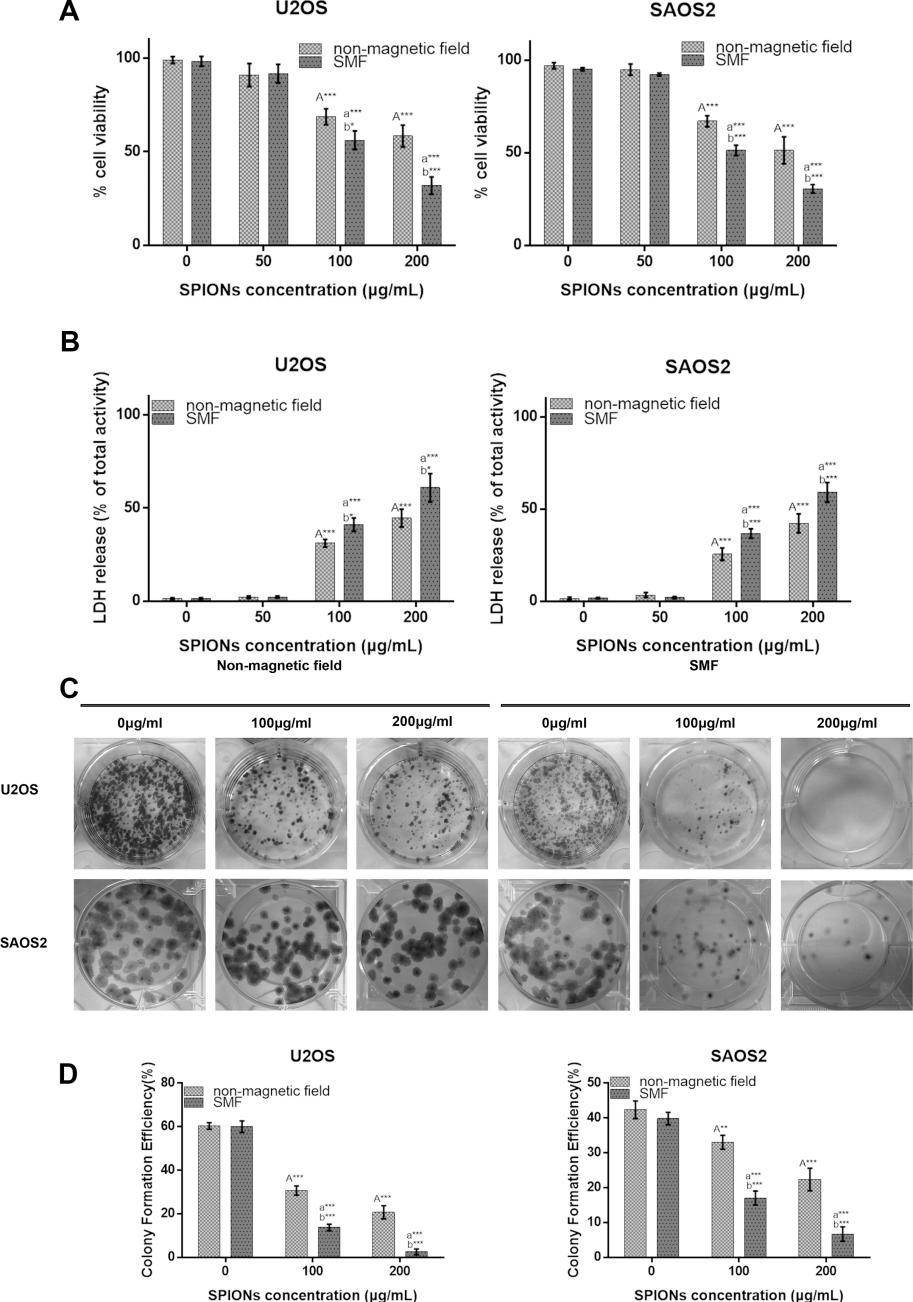
Overdose of SPIONs (≥ 100 μg/mL) induces obviously antiproliferative effect and combined with SMF exaggerates it on the osteosarcoma cell lines Cytotoxicity of non-magnetic field and SMF treated osteosarcoma cell lines by CCK-8 (**A**) and LDH assays (**B**) after incubation with SPIONs (0, 50, 100, 200 μg/mL) for 48 hours. (**C**) The colony formation assay of osteosarcoma cell lines incubated with SPIONs (0, 100, 200 μg/mL) for 15 days with the present or absent of SMF. (**D**) The statistics results of colony formation efficiency. “A,””a,” indicate compare against 0 μg/mL of SPIONs under non-magnetic field and SMF respectively; “b” compared with SPIONs treaded group of osteosarcoma cell lines with different concentration respectively. *P < 0.05, **P < 0.01, ***P < 0.001 were considered significant.

The cytotoxicity of the SPIONs in U2OS and SAOS2 cell lines was evaluated using the CCK-8 and the LDH detection in the presence/absent of SMF. The cell viability was determined by measuring the WTS-8 [2-(2-methoxy-4-nitrophenyl)-3-(4-nitrophenyl)-5-(2,4-disulfophenyl)-2H-tetrazolium, monosodium salt] to formazan, and reflected the living cells’ metabolic activity, data expressed as the percentage of the untreated control. The IC50 of SPIONs for the U2OS cells was 205.451 μg/ml in SPIONs group and 103.408 μg/ml in combination group, the IC50 for the SAOS2 cells 196.442 μg/ml in SPIONs groups and 101.057 μg/ml with combination group after incubated for 24. In this study we choose 200 μg/ml and 100 μg/ml as overdose SPIONs concentration for SPIONs group and combination groups respectively. In Figure 2A, the results indicated that the cell viability decreased as the SPIONs concentration increased. Furthermore, the cell viability showed a greater SPIONs-induced reduction in SMF than in non-magnetic field. As shown in Figure 2A, compared with cells in the absent of magnetic field, a much higher cytotoxicity was detected for the SMF present cells with SPIONs with a concentration of 100 and 200 μg/ml respectively, and both of the two concentration groups have less cell viability compared with the group treated without SPIONs, while the viability of two cell lines treated with 50 μg/ml did show significant differences.

To further investigate the SPIONs-mediated cytotoxicity on the SMF osteosarcoma cell lines, LDH released into the culture medium, which is a sensitive indicator for damage of cell membrane integrity, was evaluated after the cells were exposed to SPIONs. We found that the 100 and 200 μg/ml groups showed significant higher cytotoxicity than the non-SPIONs treated group, and compared with cells grown in non-magnetic field, a much higher cell cytotoxicity was examined for cell grown in SMF treated group with the two SPIONs concentrations (Figure 2B), which further confirmed the CCK-8 assay results.

We also performed colony formation assay simultaneously to check the treatment on the survival and proliferation of the two kinds of osteosarcoma cell lines. The results in Figure 2C demonstrated that the U2OS and SAOS2d cell lines display lower survival as the concentration of SPIONs increased, and the SMF combined with SPIONs dramatically inhibited the colony formation compared with the same concentrated SPIONs treated only group. Then quantitative changes in colony formation were determined by colony formation efficiency (Figure 2D) as previous described, and the results consistent with the results of CCK-8 and LDH assay.

### Overendocytosis of SPIONs induce apoptosis

**Figure 3:**
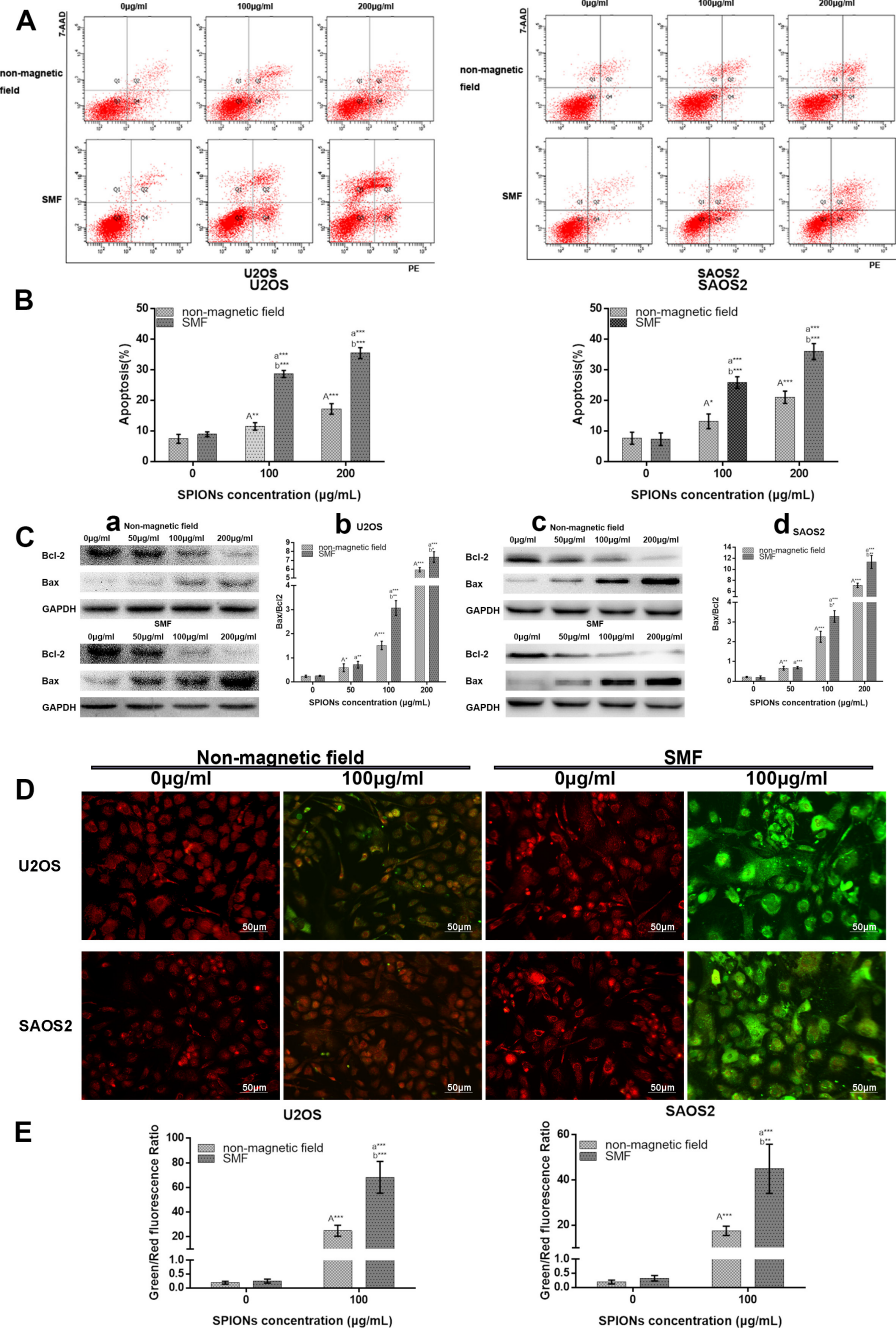
SPIONs triggers apoptosis and attenuated mitochondrial transmembrane potential FCM images (**A**) and quantitative data (**B**) of osteosarcoma cell lines exposed to 0, 100 or 200 μg/mL of SPIONs for 48 hours in non-magnetic field or SMF with Annexin V and 7AAD staining. (**C**) The Western Blot analysis of of Bax and Bcl-2 expression 48 h after treatment, C-a, -c Western blot analysis results and C-b,-d statistical analysis. (**D**) The JC-1 staining of mitochondrial depolarization of osteosarcoma cell lines treated as the Annexin V and 7AAD staining assay. (**E**) Quantitative analysis of the shift of mitochondrial orange-red fluorescence to green fluorescence among groups. “A,””a,” indicate compare against 0 μg/mL of SPIONs under non-magnetic field and SMF respectively; “b” compared with SPIONs treaded group of osteosarcoma cell lines with different concentration respectively. *P < 0.05, **P < 0.01, ***P < 0.001 were considered significant.

To further understand the underlying mechanisms of cell death, we examined the apoptosis effects. The Annexin V/7AAD double staining was employed using flow cytometry to measure apoptosis in U2OS and SAOS2 treated with/without SMF and exposed to various concentration of SPIONs. Annexin V can binds to phosphatidylserine (PS) and specifically target and identify apoptotic cells, and 7AAD can distinguish early and late stage of apoptotic cells. The percentage of apoptotic cell death was displayed as the sum of the percentage of early and late apoptotic cells. As showed in Figure 3A and 3B, there was only few apoptotic cells detected in the non-SPIONs treated groups either exposed to the non-magnetic field and SMF(8.75% ± 1.23% and 9.56% ± 0.53% for U2OS, 8.24% ± 1.56% and 7.99% ± 1.83% for SAOS2 respectively). However, when exposed to 100 μg/mL SPIONs, a significant increase of apoptotic percentage was detected, and the combination treatment of SPIONs and SMF showed more apoptotic cells (28.65% ± 0.96% for U2OS and 26.65% ± 1.23 for SAOS2), while monotherapy with SPIONs led to fewer apoptotic cells (13.75% ± 0.83% for U2OS and 14.95% ± 1.82). When it came to 200 μg/mL concentration of SPIONs, the apoptotic fraction get much higher(35.5% ± 1.19% and 33.76% ± 1.42% of SPIONs and SMF combination treatment for U2OS and SAOS2 respectively, 17.65% ± 1.36% and 18.48% ± 1.27% of SPIONs treatment only for U2OS and SAOS2 respectively). Furthermore, engulfed of SPIONs gradually caused a increase of the Bax/Bcl-2 ratio both in the two cell lines and SPIONs combined with SMF can exaggerate it significantly (Figure 3C a, b, c and d).

Moreover, we subsequently investigated the treatment effects on osteosarcoma mitochondrial membrane potential using a JC-1 mitochondrial probe. Since 100 μg/mL SPIONs combined with or without SMF could induce a significant cytotoxicity and apoptosis, we chose the concentration of 100 μg/mL to explore the next mechanisms. As shown in Figure 3D, treated with non-SPIONs cells stained with JC-1 emitted red fluorescence with a little green fluorescence. After administrate the SPIONs into cells for 48 h, the aggregated JC-1, which stayed in normal mitochondria was dispersed to monomeric form, and emitted green fluorescence. In comparison with that in the SPIONs treated only group, the treatment combination of SPIONs and SMF group further decreased mitochondrial potentials, which produced more obvious green fluorescence, and the green/red fluorescence ratio increased correspondingly (Figure 3E).

### SPIONs enhance autophagy in cells exposed to SMF

**Figure 4:**
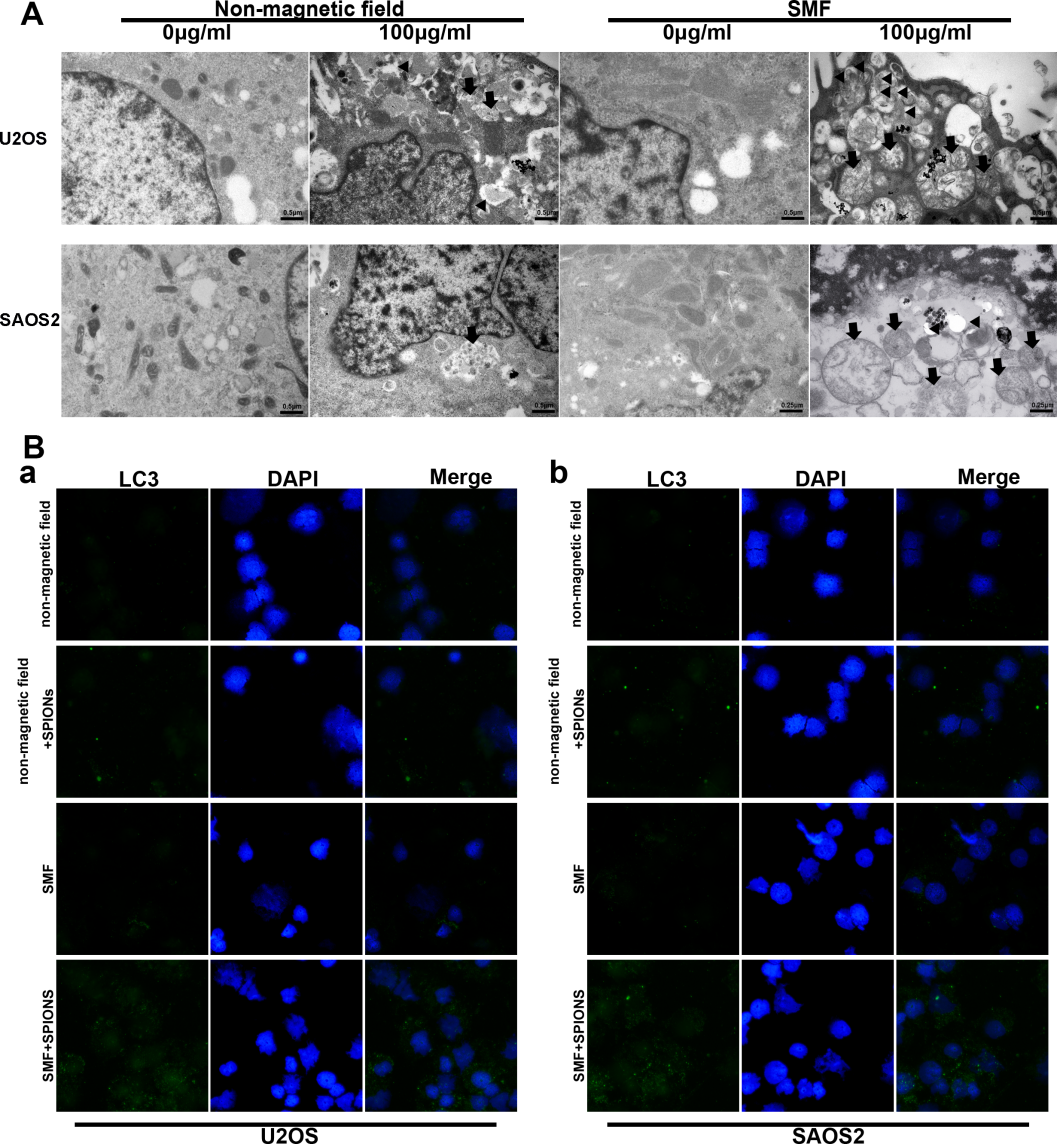
SPIONs induce apoptosis, mitochondria impair and autophagy in osteosarcoma cell lines (**A**) represent TEM images of non-magnetic field or SMF treated osteosarcoma cell lines after 48 hours incubated with different concentration of SPIONs (0, 100 μg/mL). Impaired mitochondria (black arrows) and autophagosomes (black triangle). (**B**) immunofluorescent images of the cells showing LC3B-protein marked vesicles.

Autophagy is generally considered to be a pathway for cell to survival, but it is also suggested to be a cell death mechanism. Few work has demonstrated that iron oxide nanoparticles can induce autophagy [24, 25]. According to these founding, we proceeded to investigate whether autophagy take place in SPIONs-induced cytotoxicity when cells exposed to SMF. Therefore, TEM analysis was used to detect the subcellular changes from different groups. As shown in Figure 4A, SPIONs mainly stayed in plasma and induced several mitochondria edema compared with the non-SPIONs treated cells. When combined with SMF, more impaired mitochondria was shown and the SPIONs were more prone to undergo more endocytosis, which caused the formation of a great amount of autophagosome. Thereafter, LC3 (autophagy biomarker) immunofluorescence was used to assessed the autophagy induction.

In Figure 4B a and b, the SPIONs-treated SMF exposed cells induced a significant accumulation of LC3 puncta, the SPIONs-treated only group showed fewer LC3 puncta, which in line with our previous TEM observation. To sum up, the result indicated that SPIONs could contribute to the autophagy production without SMF and the combination of SMF and SPIONs could exaggerate the effect.

### SPIONs enhance ROS generation combined with SMF

**Figure 5:**
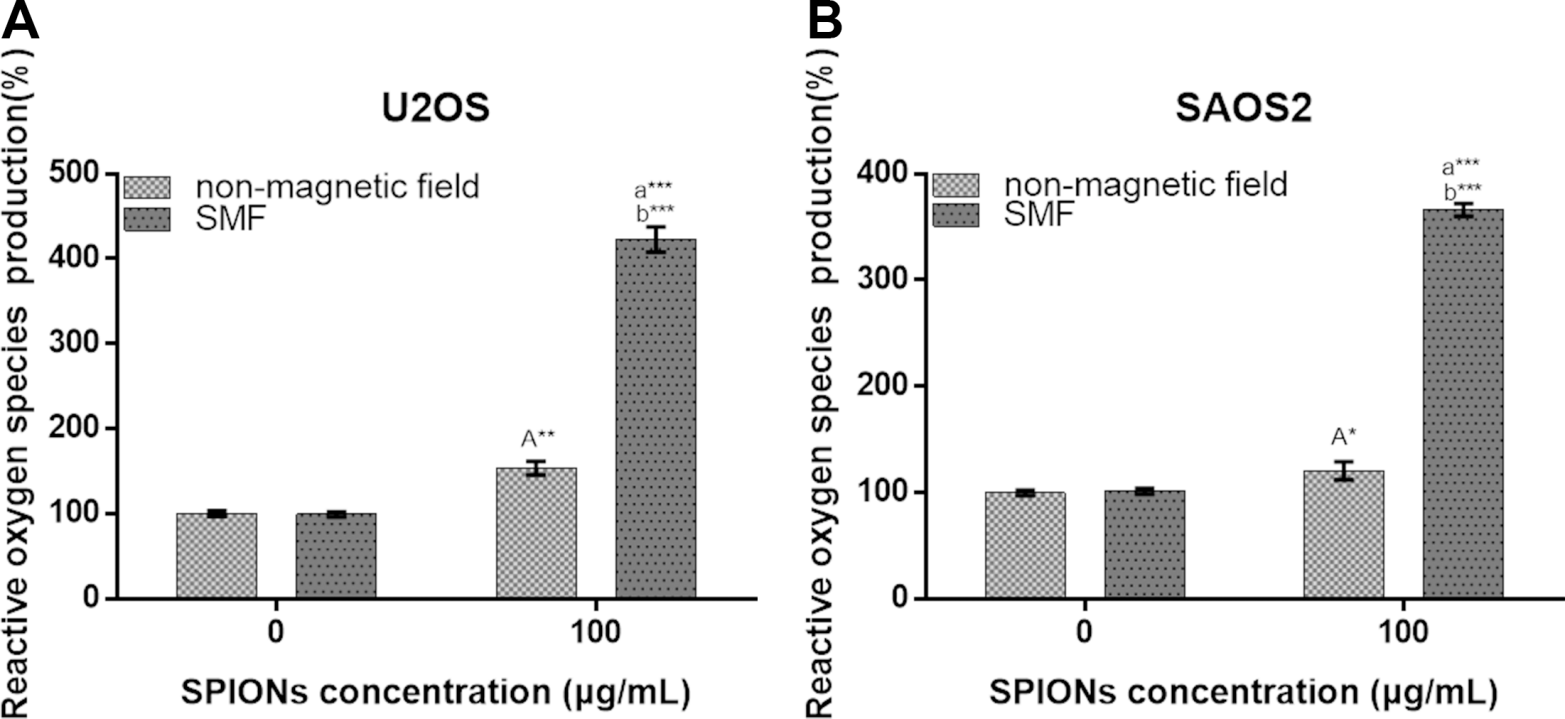
Reactive oxygen species generation of osteosarcoma cell lines Cells were treated with non-magnetic field or SMF after 48 hours incubated with 0 or 100 μg/mL SPIONs. “A,” (**A**) indicate compare against 0 μg/mL of SPIONs under non-magnetic field and SMF respectively; (**B**) compared with SPIONs treaded group of osteosarcoma cell lines with different concentration respectively. *P < 0.05, **P < 0.01, ***P < 0.001 were considered significant.

Oxidative stress plays a very important role in various vital movements of proliferation and apoptosis, and the level of intracellular ROS serve as a indicator for oxidative stress, which is essential in participate in forming autophagy. To assess the role of oxidative stress, intracellular ROS generation was examined after 48 hours of treatment with/without SPIONs in both non-magnetic field and SMF conditions. In Figure 5, the data showed that there was an approximately 1.5-fold and 1.25-fold increase in ROS in the SPIONs treated only group over the non-SPIONs and non-magnetic field treated group for U2OS and SAOS respectively, and 4.2-fold increase in the SPIONs and SMF combination group over SMF treated only group for U2OS and SAOS respectively. Therefore, the data above suggested that the SPIONs triggered increase of intracellular ROS level, and the SMF enlarged the condition.

### Study of relationship between SPIONs-induced autophagy and apoptosis

**Figure 6:**
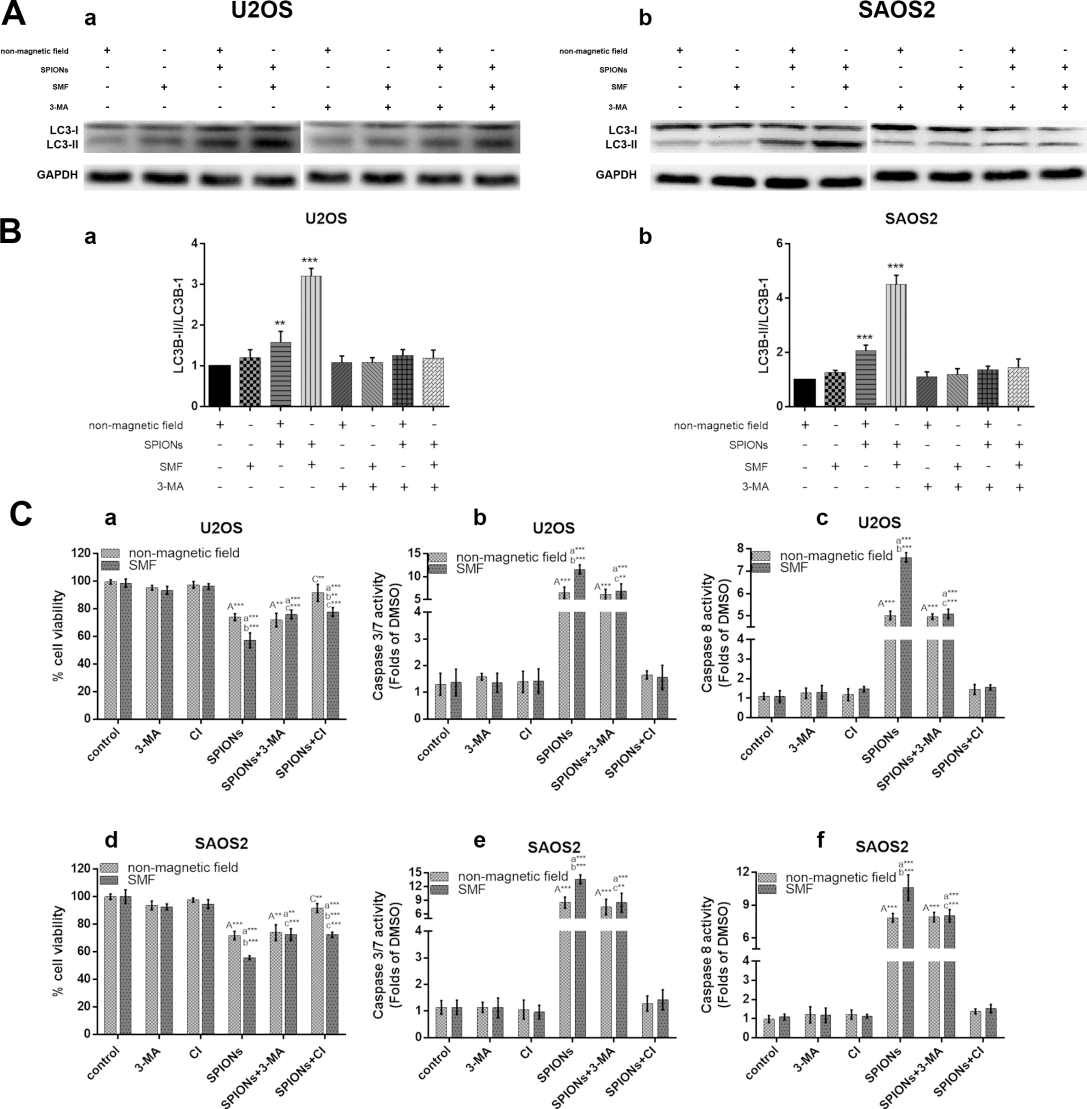
Crosstalk between the SPIONs induced apoptosis and autophagy The osteosarcoma cell lines were incubated with 0 or 100 μg/mL SPIONs for 48 hours under non-magnetic field or SMF in the presence and absence of pretreated (for 3 hours) with 3-MA and used western blot analysis to analyze autophagy proteins LC3B (**A**) and relative expression ratio of LC3-I/LC3-II (**B**) Moreover, cell pretreated (for 3 hours) and caspase inhibitor (CI, 50 μM) respectively, followed by SPIONs exposure for 48 hours to analyze cell viability (C-a, -d), caspase-3/7 expression (C-b, -e), and caspase-8 expression (C-c, -f).

To clarify the cross talk between SPIONs talk between autophagy and apoptosis, we inhibited either phenomenon to investigate the alteration in cell viability along with apoptotic or autophagy progression. We inhibited the autophagy and apoptosis with 3-methyladenine (3-MA, a autophagy inhibitor) and Ac-DEVD-CMK (a caspase inhibitor, CI, 50 μM), respectively, and determine the growth inhibitory potential by CCK-8 assay after 48 h culture. As we expected, 3-MA significantly inhibited the SPIONs/SMF induced autophagy (Figure 6A, 6B). The 3-MA pretreatment group on exposure to SPIONs/SMF (48 h) significantly increased the cell viability, the same as that of Ac-DEVD-CMK pretreated cells when compared with SPIONs/SMF treated only group (Figure 6C a, b). Additionally, caspase-3/7, 8 activity was obviously raised in SPIONs treatment group, and SPIONs/SMF treatment cell showed significantly decreased when pretreated with 3-MA, compared with SPIONs/SMF treated only group (Figure 6C b, c,d,e). Ac-DEVD-CMK could largely abrogate the caspase-3/7, 8 activities, either exposed to SPIONs or SPIONs/SMF treatment. This finding suggested that autophagy accelerate SPIONs/SMF to apoptosis, but autophagy only contribute to part of it.

### SPIONs/SMF significantly inhibits tumor formation in Xenograft mice

**Figure 7:**
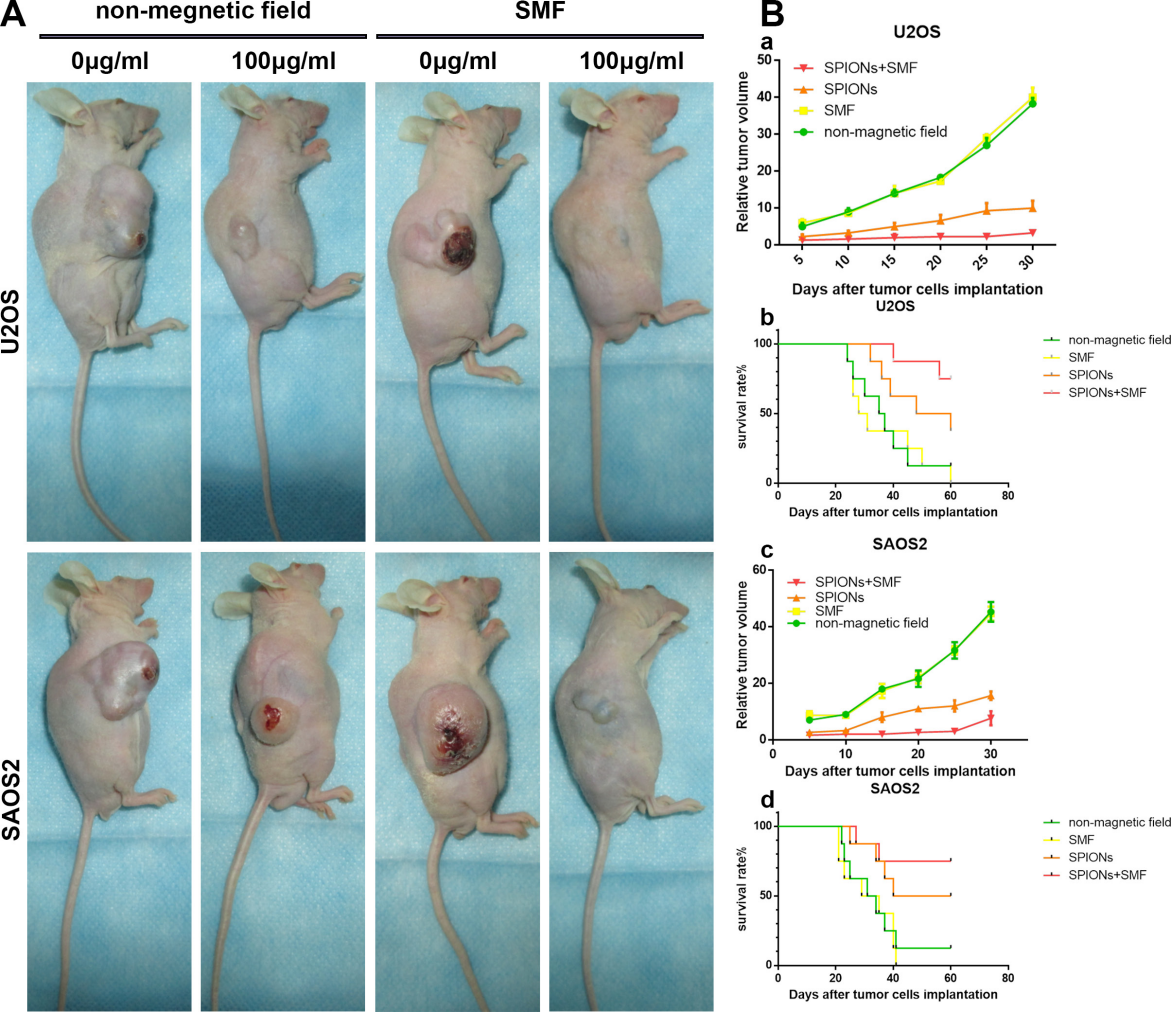
Effect of SPIONs on tumor growth in osteosarcoma cell xenograft mice (**A**) Photographs of mice 35 days after tumor cell injection (**B**-a, -c) Relative tumor volume and (B-b, -d) survival period of mice without treatment and treated by intratumor injection SPIONs, in the present or absent of SMF.

The effect of SPIONs and SPIONs/SMF combination treatment was determined in xenograft mice inoculated with U2OS and SAOS2 cell. SMF group and SPIONs combined with SMF group were administered with SMF every other day last for 30 days for 2 hours per each. We found that the diameters of tumors in the SPIONs group and SPIONs and SMF combination group were visibly smaller than the tumor of SMF or non-magnetic field treated only group (Figure 7A). In line with the previous results, the volume of the SPIONs group and SPIONs and SMF combination group were significantly lower (Figure 7B a, c), and longer lifetime and higher survival rate (Figure 7B b, d) the SMF or non-magnetic field treated only group. Moreover, the SPIONs and SMF combination group showed the weakest tumor formation ability than the other groups. These results indicated that the tumor growth was markedly inhibited by SPIONs *in vivo*, and the effects was enlarged combined with SMF, but SMF only treated group had no difference with that of non-magnetic field treated group.

## DISCUSSION

We now report for the first time a comprehensive description on the anti-tumor capacity of SPIONs both *in vitro* and *in vivo*. To be a member of nanomaterials, SPIONs are of non-chemical modification, which is different from other anti-tumor nanoparticles. Several recent studies showed that gold nanoparticles [26, 27], chitosan nanoparticles [28], and silicon nanowire [29] that killed tumor cells by apoptosis or necrosis. In this research, we documented that SPIONs provoke cytotoxicity on osteosarcoma cell lines. Our data obtained from CCK-8 and LDH assays showed a significant cytotoxic response after a 48 hours exposure to 100 μg/mL or more of SPIONs treatment groups. In addition, at the same dosage, the viability of the SMF treated group was much lower than that of the SPIONs treated only group. Which may confirm our previously expectation that SMF contributed to the increased cytotoxicity of SPIONs by physical rotation and vibration of the SPIONs.

Because the effects of the overdose SPIONs (≥ 100 ng/mL) in SMF were assured, we primarily focused attention on the potential molecular mechanisms. In this study, our results indicated that SPIONs treated cells under non-magnetic field were able to go through ROS induced apoptosis, while the SPIONs treated cells under SMF often underwent autophagic cell death and apoptosis.

Apoptosis is a pivotal way for cells to maintain homeostasis in terms of cell death and cell division, and in our study the flow cytometry analysis identified overdose SPIONs increased apoptosis in both non-magnetic field and SMF, while the later one resulted in a much higher rate of apoptosis, which is consistent with the colony formation assay.

Mitochondrial dysfunction exacerbates nanoparticle-mediated toxicity [30], induces multiple cell responses. The loss of ΔΨM can be enhanced by many key apoptosis regulators. In the present study, a significant reduction of mitochondrial potentials was shown in the SPIONs treated cells, and a higher loss was observed in the combination groups, which indicated that the cell death observed in this experiment, may originate from mitochondrial dysfunction.

The TEM analysis showed that the SPIONs accumulated around mitochondria, and there were many swollen mitochondria observed in the SPIONs treated cells, which is an evidence for the mitochondrial dysfunction as previously described. Meanwhile, vesicles of lysosomes and phagosomes were shown in the combination treatment group of SPIONs and SMF, indicating that the combination therapy can directly induced the autophagosomes formation. Moreover, multiple assays, including the autophagy biomarker of LC3 expression, conversion of LC3-I to LC3-II, and inhibition assay by 3-MA, demonstrated that SPIONs could induce autophagy in osteosarcoma cell lines, while SMF treatment could significantly enhance SPIONs-induced autophagy. To our knowledge, autophagy can removed caspases and damaged organelle, which promote cell survival; however, on the contrary, it is also observed that autophagy eventually lead to cell death. Recently, some nanoparticle types have been identified as a new type of autophagy inducers and activators of autophagic cell death [31, 32]. Yu et al [33] showed that rare element nanoparticles induce autophagy formation in human liver cells. Also, Chen et al. [31] confirmed that autophagic cell death occurred in non-small cell lung cancer cells after exposed to rare earth oxide nanoparticle. In this experiment, the role of autophagy in cell death was further confirmed by several methods. Cell pretreatment with autophagy inhibitor 3-MA raised approximately 25% of cell viability in the combination treatment group of SPIONs and SMF in U2OS and 20% in that of SAOS2. Furthermore, the percentage of caspase3/7, 8 showed a significant decrease in both cell lines, indicating that apoptosis was partly mediated by autophagy.

The autophagy in cell death and apoptosis has been proposed, but the mechanisms of the complex autophagy-apoptosis cross talk remain unclear. It is widely recognized that ROS play an essential role in the autophagy formation and cell apoptosis. Our results showed that overdose SPIONs treated cells induced amount of ROS and the phenomenon exaggerated when combined with SMF. Mitochondria are considered to be one of the major organelles that can be affected by toxicity of nanoparticle. The relationship among mitochondria and ROS production is highly closed [34]. The structural injury of mitochondria can activate an abnormal cellular ROS balance, while overexpression of ROS production can in turn induce mitochondrial damage [18]. In TEM assay, the overdose SPIONs groups showed high partial of mitochondrial impaired, and exaggerated when combined with SMF, which is in line with the ROS generation. Due to the damage of mitochondria, autophagy can be induced by ROS, therefore, mitochondria contribute to the most part of ROS for stimulating autophagy [35].

Taken together, ROS connect the two important cell reaction-apoptosis and autophagy-together, and mitochondria are the source and activator of ROS. Recently, there is a new theory that autophagosomal membrane serves as membrane scaffolds on which the caspase can combine on, and then increase the apoptosis [36]. This theory affirmed our results that with the amount of autophagy raised, caspase activity enhanced, while the cell viability decreased. However, we should also notice that, to the SPIONs and SMF treatment combination group, there was no significant differences between the 3-MA pretreated and CI pretreated subgroups in the cell viability, and both of them didn't get totally viability survived compared with the non-treatment groups, which means that there must be another mechanism for the combination therapy to contribute to these part of cell death, and we still need to further investigate.

**Figure 8:**
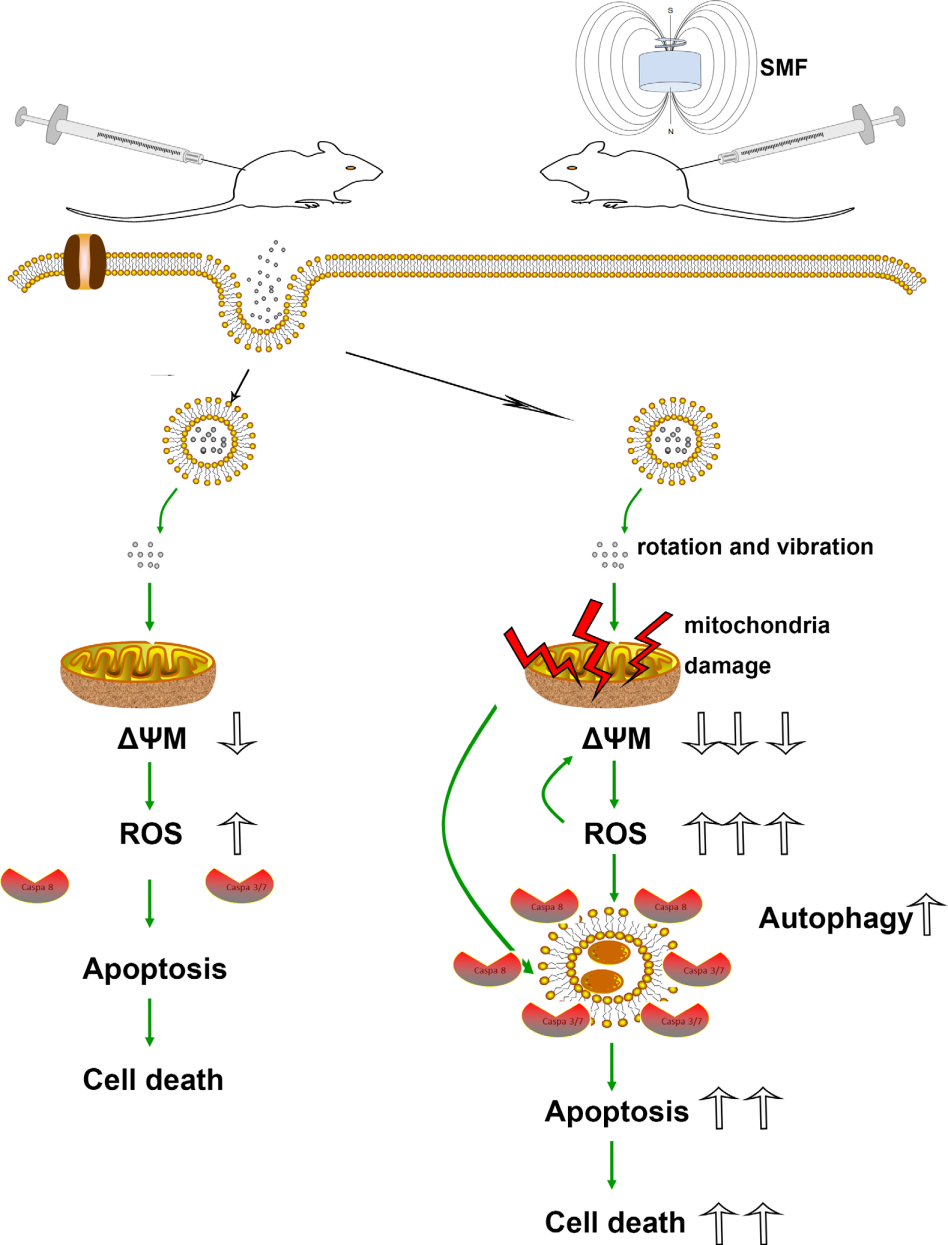
Schematic models of the effects of SPIONs combined with or without SMF in osteosarcoma cells Overdose of SPIONs induce apoptosis and cell death through loss of ΔΨM and ROS generation; however, in the SMF treatment cells, SPIONs exposure leads to the much serious loss of ΔΨM and ROS generation, meanwhile an increase in apoptosis and autophagic cell death.

Figure 8 is an overview of the SPIONs-induced cell death pathway. Briefly, overdose of SPIONs triggers the loss of ΔΨM in the early stage, which leads to mitochondrial dysfunction and ROS production. However, combined with SMF treatment, the SPIONs physical rotation and vibration under which can directly impair the organelle, especially the mitochondria. With the loss of ΔΨM drastic decrease and ROS production increase, the membrane of autophagy formation provide caspase a platform to react, which exaggerate the apoptosis and cell death.

In sum, we have clearly demonstrated that overdose SPIONs lead to mitochondrial dysfunctions and osteosarcoma cells apoptosis; SPIONs under SMF trigger the autophagy formation and exacerbate the autophagic death and apoptosis.

## MATERIALS AND METHODS

### Synthesis and characterization of SPIONs

SPIONs with an average particle size of 18 nm were produced via a co-precipitation method of ferric salts and ferrous according as previously described [11, 12]. In brief, 25 mL aqueous solution of 6.8 mmol FeCl_3_ (Sigma Aldrich, USA), 4.3 mmol FeCl_2_·4H_2_O (Sigma Aldrich, USA) served as a source of iron. The magnetite particles were coprecipitated under vigorous mechanical stirring by dropwise adding 4ml 25% ammonia aqueous solution (NH_3_· H_2_O) (Sigma Aldrich, USA), then after 30 min, a PVA solution (polymer to iron mass ratio of 2; this amount of polymer is able to create non-aggregated coated particles [16]) was added as a stabilizer, and the reaction was then allowed to proceed for an additional 30 min. Then a N_2_ atmosphere was carried out to prevent oxidation for another 30 min. The particles were collected by a permanent magnet and were wash three times with degassed water and then the precipitates were filtered through a 0.22 μm filter and kept at 4°C.

Transmission electron microscope (TEM) (Hitachi H-9500, Japan), X-ray diffraction (XRD) (UltimaIV, Rigaku, Japan) and vibrating sample magnetometer (VSM) (Lakeshose 7404, Quantum Design, USA) was carried out to evaluate the physical property of the nanoparticles. For TEM analysis, on drop of suspension of magnetite nanoparticle dispersed in EtOH was placed on a carbon-coated copper grid and evaporated to dry. XRD measurement was performed using CuK_α_ radiation (λ1.5406Å) at 30 mA and 30 kV. VSM was performed on Lakeshose 7404 magnetometer operating at ambient temperature.

### Cell culture

The human osteosarcoma cell U2OS and SaOS-2 cell lines were stored in our laboratory and the original source of the two cell lines were procured from Cell Collection of Chinese Academy of Science (Shanghai, China). U2OS were cultured in in RPMI 1640 medium (Gibco, Rockville, USA) and SaOS-2 were grown Dulbecco's modified Eagle's medium (DMEM) (Gibco) respectively. All media were supplemented with 100 μg/ml penicillin-streptomycin, 10% fetal bovine serum (FBS, Invitrogen, Carlsbad, USA). Cell were maintianed in a humidified incubator containing 5% CO_2_ at 37°C.

### The spinning magnetic field application

The spinning magnetic field (SMF) application used in this study was a cylinder that spin on its long axial, the spinning speed is 300 rpm and magnetic field strength is 8 kA/m. The cylinder magnetic field application was a permanent magnet.

### Cell viability assay

Cell counting kit-8(CCK-8) assay was applied to evaluate the *in vitro* cytotoxicity of SPIONs and SMF on Osteosarcoma cells. The two Osteosarcoma cell lines were plated in triplicate into 96-well plate at a final density of 1 × 10^4^ (U2OS) or 5 × 10^3^ (SaOS-2) cells/well in 200 μl medium and were incubated for 24 h. After 24 h, the cells were subsequently treated with SPIONs at increasing concentrations (50 μg, 100 μg, 200 μg/ml) and the control medium with or without SMF. For the magnetic field treatment group, the cell exposed to magnetic field for 3 h per day. Thereafter, the supernatant was removed and CCK-8 was added to measure the percentage of survived cells according to the manufacturer's instructions and qualified the optical density at 450 nm using a microplate reader.

### LDH release

Cytotoxicity was detected by quantifying the activity of lactate dehydrogenase (LDH) released into culture medium. LDH activities of medium relative to total LDH activity represent the percentages of injured cells in cultures after complete cell lysis. In briefly, take a portion of culture medium to react with an equal volume of LDH substrate solution for 30 min. Stopped by adding 5 volume of 0.1 M NaOH, the absorbance was then measured in a microplate reader at 440 nm. sister culture were treated using 1/100 volume of 10% Triton X-100 and incubated at 37°C for 30 min. medium containing Triton-lysed supernatant were used to determine the total LDH activities.

### Colony formation assay

The influence of SPIONs and SMF on the reproductive potential of osteosarcoma cells was assessed by colony formation assays. Briefly, osteosarcoma cells were plated in triplicate at a density of 400 cels/well in six well plates. After adherence for 24 h, medium with different concentration of SPIONs were added, and for another 48 h culture, the supernatant was removed, and all wells were rinsed with PBS softly twice to remove the SPIONs that were not engulfed by cells. Thereafter, normal medium were add back. The SMF was applied for the magnetic field treatment group for 3 h per day. After culture for 15 days, the colonies were then fixed in methanol for 10 minutes and stained with crystal violet for 10 minutes at room temperature. The number of colonies were counted using an inverted microscope.

### Annexin V and 7AAD staining

Osteosarcoma cells were treated with or without SMF field at different concentration of SPIONs in 6-well plate. Then cells pellets were collected by trypsinization. washed with PBS and centrifuging at 200 g for 5 min. To assess the percentages of apoptotic and viable cells, double-staining of using annexin-V coupled with Phycoerythrin (PE)/7-AAD was performed using the Annexin V-PE/7-AAD Apoptosis Detection Kit (BD Biosciences, USA) according to the manufacturer's instructions and analyzed by flow cytometry.

### Mitochondrial transmembrane potential assay

Loss of mitochondrial transmembrane potential (ΔΨm) was analyzed by a fluorescent probe JC-1 (Beyotime, Jiangsu, China), following the manufacturer's protocol. Briefly, cells in 6-wells plates after indicated treatments were stained with an equal volume of JC-1 staining solution (20 μg/ml) for 15 min at 37°C in dark and washed twice with PBS. Thereafter, images were captured under an Olympus fluorescent microscope. JC-1 forms as J-aggregates in the mitochondrial matrix and give a red fluorescence when ΔΨm is high. After the mitochondrial membrane depolarization, the JC-1 appears in the cytosol as monomers and emits green fluorescence. The ratio of green/red fluorescence intensity was used to indicate the mitochondrial depolarization.

### TEM analysis of subcellular ultrastructure and SPIONs localization

TEM analysis was performed to assess the intracellular localization of the SPIONs and the morphology changes of the subcellular ultrastructure. After treated in 6-well plate, cells were collected by a cell scraper, pelleted using centrifugation and fixed in 2.5% glutaraldehyde for 12 hours at 4°C. Post-fixation with 1% osmium tetroxide was performed for 2 hours at room temperature, followed by dehydrated with a graded series of alcohols and 100% acetone before embedding the pellets spur resin. Ultrathin sections (50∼70 nm) was cut and placed onto copper grids. The ultrastructural analysis was examined under Hitachi H-7650 TEM (Japan).

### Immunofluorescence and confocal imaging

Cell grown on cover glasses were treated with various concentration of SPIONs with or without SMF as designed. After 24 hours, the cells were rinsed with PBS, fixed in 4% paraformaldehyde and permeabilized by 03% Triton X-100. After blocking in 3% bovine serum albumin, the cells were serially incubated in rabbit anti-LC3B (3868S, Cell Signaling Technology, Inc. USA, 1:200) at 4°C overnight and Goat anti-rabbit Alexa Fluo488 (Invitrogen, 1:3000) for 1 hour at room temperature in the dark. Then the cells were stained with nuclear stain DAPI (10 μg/mL) for 5 minutes at room temperature and the image was observed under a confocal microscope (FV 1000; Olympus Corporation, Tokyo, Japan).

### ROS generation determination

The levels of intracellular reactive oxidant species (ROS) was based on the oxidative conversion of 2, 7-dichlorodihydrofluorescein diacetate (DCFH-DA) (Beyotime Biotech) to fluorescent dichlorofluorescein (DCF) in a microplate reader (tecan infinite 200) as previously described [37]. Briefly, osteosarcoma cells were plated in 96-well plate in triplicate and treated with indicated concentration of SPIONs in the presence or absence of SMF as mentioned in CCK-8 assay. Thereafter, cells were washed with PBS and incubated with 100 μM DCFH-DA in dark at 37°C for 30 min. Then the fluorescence distribution of the cells from each well was detected by the fluorescence microplate reader at the excitation wavelength and emission wavelength set as 488 nm and 535 nm respectively.

### Western blot analysis

For the analysis of bcl2, bax, LC3B, osteosarcoma cells were resuspended in a protein lysis buffer RIPA (Boster bio, Wuhan, China) containing protease inhibitors (Boster Bio, Wuhan, China) and phosphatase inhibitor (Boster Bio, Wuhan, China) at 4°C for 30 min incubation. After homogenization, the supernatants containing protein was collected by centrifugation and stored at –80°C. Followed by the BCA protein assay, protein samples were separated by 10%–12% SDS-PAGE electrophoretically and were then transferred to a PVDF membrane (GE Healthcare life Sciences, Piscataway, USA). Then western blotting were proceeded with primary and secondary antibodies and was detected with peroxidase-conjugated anti-rabbit antibody followed by enhanced chemiluminescence (Amersham Pharmacia Biotech, Piscataway, USA). The details of the primary antibodies and dilution factors were as follows: Bcl2 (Cell Signaling Technology, Inc. USA, 1:1000), Bax (Cell Signaling Technology, Inc. USA, 1:1000), LC3B (Cell Signaling Technology, Inc. USA, 1:1000), GAPDH (Cell Signaling Technology, Inc. USA, 1:1500).

### Tumor xenograft studies

This study was approved by the ethical committee of animal experiment in Zhejiang University, and animal procedures performed were in accordance with the Public Health Service policy. Male BALB/c-nude mice were kept in a room with controlled temperature and light, provided with sterile water and chow. After being acclimated for a week, nude mice were distributed in 9 groups randomly: normal (*n* = 8), U2OS xenograft (*n* = 8), U2OS xenograft-SMF (*n* = 8), U2OS xenograft-SPIONs (*n* = 8), U2OS xenograft-SPION/SMF (*n* = 8), SAOS2 xenograft (*n* = 8), SAOS2 xenograft-SMF (*n* = 8), SAOS2 xenograft-SPIONs (*n* = 8), SAOS2 xenograft-SPION/SMF (*n* = 8). The mice were inoculate with U2OS or SAOS2 cells by intradermal injection (7 × 10^6^ cells/0.2Ml/mouse) with a 27-gauge needle into the flank to establish tumor xenograft. The treatment began on 4 days after the injection when the average tumor diameter reached 7 mm. The mice in the SPIONs and SPIONs/SMF treatment groups were injected intratumorally with SPIONs at a dose of 2 mg/kg every other day for 3 times, whereas the other groups injected with 2% glucose solution at the same volume as the SPIONs. SPIONs were dispersed homogeneously in a 2% glucose solution. The SMF treatment took place for 2 hours every two days after the first day SPIONs was injected. Mouse tumor volumes were measured periodically. The tumor volume was assessed with vernier calipers according to the formula (1)

Larger dimension × (Smaller dimension)^2^ × 0.52 [38]. (1)

The number of survival rate was recorded every day and all observers were blinded in the studies related to survival rates and tumor area.

### Statistics analysis

Data are representative as mean ± S.D. of at least three independent experiments. Student's *t* test was used for analyzing statistical differences between groups. One-way analysis of variance was used for differences among groups, with Dunn's multiple group comparison tests as appropriate. SPSS Statistics 20 (IBM SPSS software, Chicago, IL, USA) and GraphPad Prism 6 (GraphPad Software, Inc., San Diego, USA) were used to do the statistical analysis. *P* > 0.05 was defined as not significant, *P < 0.05; **P < 0.01; *** *P <* 0.001 were considered significant statistically.

## References

[1] Williams DF (2009). On the nature of biomaterials. Biomaterials.

[2] Jha RK, Jha PK, Chaudhury K, Rana SV, Guha SK (2014). An emerging interface between life science and nanotechnology: present status and prospects of reproductive healthcare aided by nano-biotechnology. Nano Rev.

[3] Buzea C, Pacheco II, Robbie K (2007). Nanomaterials and nanoparticles: sources and toxicity. Biointerphases.

[4] Lewinski N, Colvin V, Drezek R (2008). Cytotoxicity of nanoparticles. Small.

[5] Chen H, Wang L, Yu Q, Qian W, Tiwari D, Yi H, Wang AY, Huang J, Yang L, Mao H (2013). Anti-HER2 antibody and ScFvEGFR-conjugated antifouling magnetic iron oxide nanoparticles for targeting and magnetic resonance imaging of breast cancer. Int J Nanomedicine.

[6] Ghazani AA, Pectasides M, Sharma A, Castro CM, Mino-Kenudson M, Lee H, Shepard JA, Weissleder R (2014). Molecular characterization of scant lung tumor cells using iron-oxide nanoparticles and micro-nuclear magnetic resonance. Nanomedicine.

[7] Gupta AK, Gupta M (2005). Synthesis and surface engineering of iron oxide nanoparticles for biomedical applications. Biomaterials.

[8] Buyukhatipoglu K, Chang R, Sun W, Clyne AM (2010). Bioprinted nanoparticles for tissue engineering applications. Tissue Eng Part C Methods.

[9] Halavaara J, Tervahartiala P, Isoniemi H, Höckerstedt K (2002). Efficacy of sequential use of superparamagnetic iron oxide and gadolinium in liver MR imaging. Acta Radiol.

[10] Bonnemain B (1998). Superparamagnetic agents in magnetic resonance imaging: physicochemical characteristics and clinical applications. A review. J Drug Target.

[11] Liu Z, Lammers T, Ehling J, Fokong S, Bornemann J, Kiessling F, Gatjens J (2011). Iron oxide nanoparticle-containing microbubble composites as contrast agents for MR and ultrasound dual-modality imaging. Biomaterials.

[12] Brismar TB, Grishenkov D, Gustafsson B, Harmark J, Barrefelt A, Kothapalli SV, Margheritelli S, Oddo L, Caidahl K, Hebert H, Paradossi G (2012). Magnetite nanoparticles can be coupled to microbubbles to support multimodal imaging. Biomacromolecules.

[13] Babincová M, Leszczynska D, Sourivong P, Babinec P (2000). Selective treatment of neoplastic cells using ferritin-mediated electromagnetic hyperthermia. Med Hypotheses.

[14] Hilger I, Hiergeist R, Hergt R, Winnefeld K, Schubert H, Kaiser WA (2002). Thermal ablation of tumors using magnetic nanoparticles: an in vivo feasibility study. Invest Radiol.

[15] Xu C, Sun S (2013). New forms of superparamagnetic nanoparticles for biomedical applications. Adv Drug Deliv Rev.

[16] Petri-Fink A, Chastellain M, Juillerat-Jeanneret L, Ferrari A, Hofmann H (2005). Development of functionalized superparamagnetic iron oxide nanoparticles for interaction with human cancer cells. Biomaterials.

[17] Yokoyama T, Tam J, Kuroda S, Scott AW, Aaron J, Larson T, Shanker M, Correa AM, Kondo S, Roth JA, Sokolov K, Ramesh R (2011). EGFR-targeted hybrid plasmonic magnetic nanoparticles synergistically induce autophagy and apoptosis in non-small cell lung cancer cells. PLoS One.

[18] Nel A, Xia T, Mädler L, Li N (2006). Toxic potential of materials at the nanolevel. Science.

[19] Kroemer G, Mariño G, Levine B (2010). Autophagy and the integrated stress response. Mol Cell.

[20] Mizushima N, Levine B, Cuervo AM, Klionsky DJ (2008). Autophagy fights disease through cellular self-digestion. Nature.

[21] RLC M Yamaura, Sampaio LC (2004). Preparation and characterization of (3-aminopropyl) triethoxysilane-coated magnetite nanoparticles.

[22] RM Conell US. (1996). The iron oxides: structure, properties, reactions, occurrence and uses. Verlagsgesellschaft mbH (VHC).

[23] Klug HP, ANA LE (1962). X-ray diffraction procedures for polycrystalline and amorphous materials.

[24] Shi M, Cheng L, Zhang Z, Liu Z, Mao X (2015). Ferroferric oxide nanoparticles induce prosurvival autophagy in human blood cells by modulating the Beclin 1/Bcl-2/VPS34 complex. Int J Nanomedicine.

[25] Tseng TC, Hsieh FY, Hsu SH (2016). Increased cell survival of cells exposed to superparamagnetic iron oxide nanoparticles through biomaterial substrate-induced autophagy. Biomater Sci.

[26] Park J, Wrzesinski SH, Stern E, Look M, Criscione J, Ragheb R, Jay SM, Demento SL, Agawu A, Licona LP, Ferrandino AF, Gonzalez D, Habermann A (2012). Combination delivery of TGF-β inhibitor and IL-2 by nanoscale liposomal polymeric gels enhances tumour immunotherapy. Nat Mater.

[27] Yao L, Daniels J, Danniels J, Moshnikova A, Kuznetsov S, Ahmed A, Engelman DM, Reshetnyak YK, Andreev OA (2013). pHLIP peptide targets nanogold particles to tumors. Proc Natl Acad Sci USA.

[28] Guan M, Zhou Y, Zhu QL, Liu Y, Bei YY, Zhang XN, Zhang Q (2012). N-trimethyl chitosan nanoparticle-encapsulated lactosyl-norcantharidin for liver cancer therapy with high targeting efficacy. Nanomedicine.

[29] Park GS, Kwon H, Kwak DW, Park SY, Kim M, Lee JH, Han H, Heo S, Li XS, Lee JH, Kim YH, Lee JG, Yang W (2012). Full surface embedding of gold clusters on silicon nanowires for efficient capture and photothermal therapy of circulating tumor cells. Nano Lett.

[30] Ding F, Li Y, Liu J, Liu L, Yu W, Wang Z, Ni H, Liu B, Chen P (2014). Overendocytosis of gold nanoparticles increases autophagy and apoptosis in hypoxic human renal proximal tubular cells. Int J Nanomedicine.

[31] Chen Y, Yang L, Feng C, Wen LP (2005). Nano neodymium oxide induces massive vacuolization and autophagic cell death in non-small cell lung cancer NCI-H460 cells. Biochem Biophys Res Commun.

[32] Zabirnyk O, Yezhelyev M, Seleverstov O (2007). Nanoparticles as a novel class of autophagy activators. Autophagy.

[33] Yu L, Lu Y, Man N, Yu SH, Wen LP (2009). Rare earth oxide nanocrystals induce autophagy in HeLa cells. Small.

[34] Yu KN, Yoon TJ, Minai-Tehrani A, Kim JE, Park SJ, Jeong MS, Ha SW, Lee JK, Kim JS, Cho MH (2013). Zinc oxide nanoparticle induced autophagic cell death and mitochondrial damage via reactive oxygen species generation. Toxicol In Vitro.

[35] Chen Y, Gibson SB (2008). Is mitochondrial generation of reactive oxygen species a trigger for autophagy. Autophagy.

[36] MM Young YT, O Khan SP, Hori T (2012). Autophagosomal membrane serves as platform for intracellular death-inducing signaling complex (iDISC)-mediated caspase-8 activation and apoptosis.

[37] Wang H, Joseph JA (1999). Quantifying cellular oxidative stress by dichlorofluorescein assay using microplate reader. Free Radic Biol Med.

[38] Davol PA, Frackelton AR (1999). Targeting human prostatic carcinoma through basic fibroblast growth factor receptors in an animal model: characterizing and circumventing mechanisms of tumor resistance. Prostate.

